# Role of preoperative air-bone gap in tinnitus outcome after tympanoplasty for chronic otitis media with tinnitus^☆^

**DOI:** 10.1016/j.bjorl.2017.01.003

**Published:** 2017-02-14

**Authors:** Hong Chan Kim, Chul Ho Jang, Young Yoon Kim, Jong Yuap Seong, Sung Hoon Kang, Yong Beom Cho

**Affiliations:** Chonnam National University Medical School, Department of Otolaryngology, Gwangju, South Korea

**Keywords:** Tinnitus, Tympanoplasty type I, Preoperative air-bone gap, Zumbido, Timpanoplastia tipo I, *Gap* aéreo-ósseo pré-operatório

## Abstract

**Introduction:**

Previous reports indicated that middle ear surgery might partially improve tinnitus after surgery. However, until now, no influencing factor has been determined for tinnitus outcome after middle ear surgery.

**Objective:**

The purpose of this study was to investigate the association between preoperative air-bone gap and tinnitus outcome after tympanoplasty type I.

**Methods:**

Seventy-five patients with tinnitus who had more than 6 months of symptoms of chronic otitis media on the ipsilateral side that were refractory to medical treatment were included in this study. All patients were evaluated through otoendoscopy, pure tone/speech audiometer, questionnaire survey using the visual analog scale and the tinnitus handicap inventory for tinnitus symptoms before and 6 months after tympanoplasty. The influence of preoperative bone conduction, preoperative air-bone-gap, and postoperative air-bone-gap on tinnitus outcome after the operation was investigated.

**Results and conclusion:**

The patients were divided into two groups based on preoperative bone conduction of less than 25 dB (*n* = 50) or more than 25 dB (*n* = 25). The postoperative improvement of tinnitus in both groups showed statistical significance. Patients whose preoperative air-bone-gap was less than 15 dB showed no improvement in postoperative tinnitus using the visual analog scale (*p* = 0.889) and the tinnitus handicap inventory (*p* = 0.802). However, patients whose preoperative air-bone-gap was more than 15 dB showed statistically significant improvement in postoperative tinnitus using the visual analog scale (*p* < 0.01) and the tinnitus handicap inventory (*p* = 0.016). Postoperative change in tinnitus showed significance compared with preoperative tinnitus using visual analog scale (*p* = 0.006). However, the correlation between reduction in the visual analog scale score and air-bone-gap (*p* = 0.202) or between reduction in tinnitus handicap inventory score and air-bone-gap (*p* = 0.290) was not significant. We suggest that the preoperative air-bone-gap can be a predictor of tinnitus outcome after tympanoplasty in chronic otitis media with tinnitus.

## Resumo

**Introdução:**

Relatos anteriores indicaram que a cirurgia no ouvido médio pode melhorar parcialmente o zumbido após a cirurgia. No entanto, até agora, nenhum fator influenciador foi determinado para o resultado do zumbido após cirurgia de ouvido médio.

**Objetivo:**

O objetivo desse estudo foi investigar a associação entre o *gap* aéreo-ósseo pré-operatório e o desfecho do zumbido após timpanoplastia do tipo I.

**Método:**

Setenta e cinco pacientes com zumbido, com mais de 6 meses de sintomas de otite média crônica no lado ipsilateral que eram refratários ao tratamento médico foram incluídos nesse estudo. Todos os pacientes foram avaliados através de otoendoscopia, audiometria tonal/vocal, questionário utilizando a escala visual analógica e o questionário *tinnitus handicap inventory* para sintomas de zumbido antes e 6 meses após a timpanoplastia. A influência da condução óssea pré-operatória, *gap* aéreo-ósseo pré-operatório e pós-operatório sobre o desfecho do zumbido após a operação foi analisada.

**Resultados e conclusão:**

Os pacientes foram divididos em dois grupos com base na condução óssea pré-operatória de menos de 25 dB (n = 50) ou mais de 25 dB (n = 25). A melhora do zumbido pós-operatória em ambos os grupos mostrou significância estatística. Pacientes com *gap* aéreo-ósseo pré-operatório inferior a 15 dB não apresentaram melhora no zumbido pós-operatório utilizando a escala visual analógica (*p* = 0,889) e *tinnitus handicap inventory* (*p* = 0,802). Entretanto, pacientes com *gap* aéreo-ósseo pré-operatório maior que 15 dB apresentaram melhora estatisticamente significante no zumbido pós-operatório utilizando a escala visual analógica (*p* < 0,01) e o *tinnitus handicap inventory* (*p* = 0,016). A mudança pós-operatória no zumbido mostrou significância em comparação com o zumbido pré-operatório usando a escala visual analógica (*p* = 0,006). No entanto, a correlação entre a redução no escore da escala visual analógica e *gap* aéreo-ósseo (*p* = 0,202) ou entre a redução no escore do *tinnitus handicap inventory* e *gap* aéreo-ósseo (*p* = 0,290) não foi significativa. Sugerimos que o *gap* aéreo-ósseo pré-operatório possa ser um preditor de desfecho do zumbido após timpanoplastia em otite média crônica com zumbido.

## Introduction

Tinnitus is the perception of noise in the ears which can take many forms such as ringing, roaring, buzzing, hissing, and others. Despite thorough and extensive research, the cause of tinnitus is yet to be determined. The prevalence of tinnitus is significantly higher among hearing-impaired persons than in the normal-hearing population.^1^ Surveys have revealed that while 10%–15% of the adult population as a whole suffers from tinnitus, as many as 70%–85% of the hearing impaired population report tinnitus.^1^, ^2^

A temporary or permanent decrease in auditory stimuli (sensory deficit) increases the sensitivity of subcortical neurons, resulting in the plastic reorganization of the auditory cortex, with subsequent sustained awareness of tinnitus.^3^ Studies on plasticity have suggested that an increase in the auditory stimulus provided by external sound amplification through the masking effect can induce secondary plasticity, helping to decrease the discomfort associated with tinnitus.^4^ There is a significant correlation between tinnitus and hearing loss in 85%–96%.^5^ Therefore, restoration of hearing by surgery or amplification by hearing aid can attenuate tinnitus. Tinnitus is a common problem in patients with chronic otitis media (COM).^6^, ^7^ Since the effect of tympanoplasty on tinnitus had been suggested by Helm for the first time,^8^ there have been few studies to date investigating tinnitus outcomes after middle ear surgery for chronic simple otitis media with tinnitus.^6^, ^7^, ^8^, ^9^ Previous reports indicated that middle ear surgery might partially improve tinnitus after surgery. However, until now, no influencing factor has been determined for tinnitus outcome after middle ear surgery. The purpose of this study was to investigate the association between preoperative air-bone gap (ABG) and tinnitus outcome after tympanoplasty type I.

## Methods

This retrospective study was conducted in patients with COM with subjective tinnitus on the ipsilateral side who underwent tympanoplasty type I under local anesthesia between January 2014 and October 2015, all performed by a single senior surgeon with the same technique (underlay) using temporalis fascia or perichondrium at a tertiary university hospital. This study was approved by the institutional review board. In total, 75 patients with tinnitus who had more than 6 months of symptoms of COM on the ipsilateral side that were refractory to medical treatment were included in the study. They were followed up for at least 6 months postoperatively. An intact epithelized neodrum without retraction or lateralization was considered a success. All patients were evaluated through otoendoscopy, pure tone/speech audiometer, questionnaire survey using visual analog scale (VAS) and tinnitus handicap inventory (THI) for tinnitus symptoms before and 6 months after tympanoplasty.

For VAS, we asked patients to assign a 0–10 score to their tinnitus, with the help of a standard scale commonly used for indicating pain level. The assessment focused on the intensity and disturbance. VAS is easily applicable and understood by most patients. THI strengthens the evaluation of the functional, emotional, and catastrophic reactions to tinnitus. Audiological evaluation by pure tone audiometry was conducted prior to tympanoplasty and 6 months after surgery. The Pure Tone Average (PTA) and ABG at 0.5, 1, 2, and 4 kHz were evaluated. Subjective attenuation of tinnitus in 75 patients was investigated before and after tympanoplasty type I. The influence of preoperative Bone Conduction (BC), preoperative air-bone-gap, and postoperative air-bone-gap on tinnitus outcome after the operation was investigated. Due to lack of follow-up audiogram in 20 patients, the relationship between reduction in ABG and tinnitus improvement was investigated in 55 patients. The statistical analyses were performed by paired *t* test and Pearson correlation test using SPSS software.

## Results

**Figure 1:**
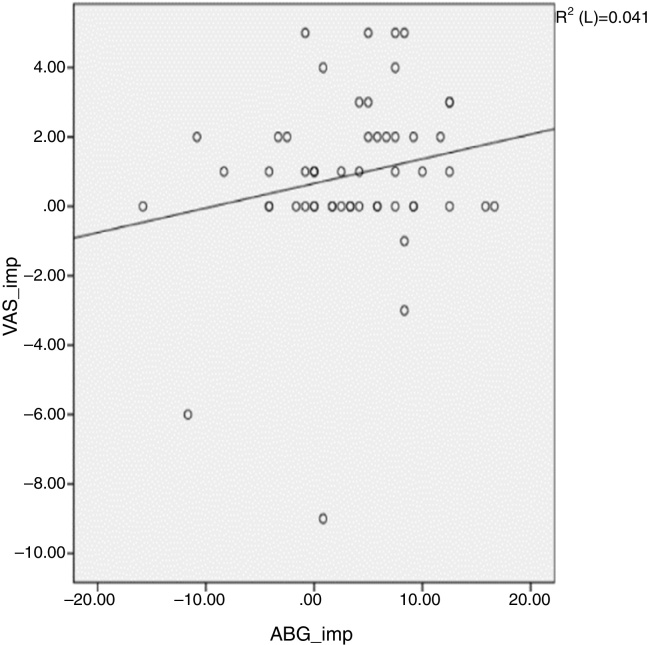
Shows an insignificant correlation between tinnitus improvement by visual analog scale (VAS) and hearing gain. Pearson's correlation coefficient *p* = 0.139.

**Figure 2:**
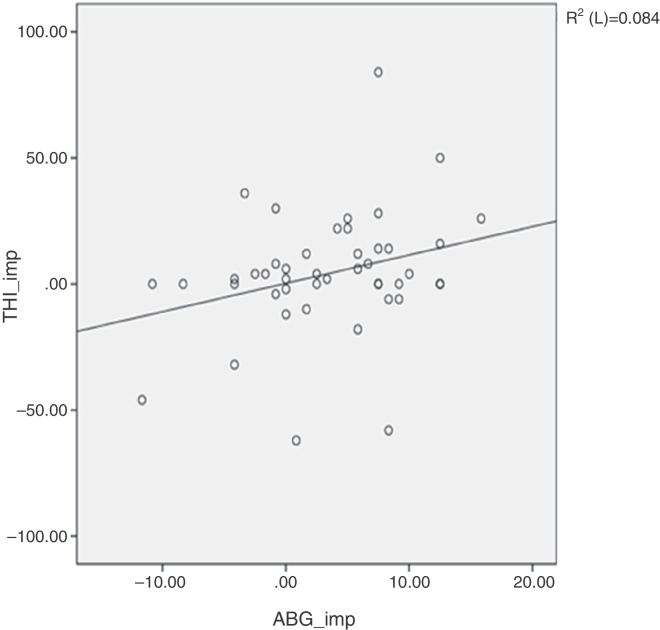
Shows an insignificant correlation (Pearson) between tinnitus improvement by tinnitus handicap inventory (THI) and hearing gain. Pearson's correlation coefficient *p* = 0.054.

The mean age of patients was 50.7 (11–78) years, with sex distribution of 24 men and 51 women. COM with tinnitus was either right sided (41 cases) or left sided (34 cases) (Table 1). Table 2 shows the size of perforation and location type. The moderate anterior perforation was most common. The mean duration of tinnitus was 29.55 months. The relationship between preoperative BC level and improvement of tinnitus was evaluated by VAS. The patients were divided into two groups based visual analog scale on preoperative bone conduction of less than 25 dB (*n* = 50) or more than 25 dB (*n* = 25). The postoperative improvement of tinnitus in both groups showed statistical significance (Table 3). The relationship between preoperative ABG and improvement of tinnitus showed different results depending on the degree of preoperative ABG (less or more than 15 dB). The subjective improvement was examined by VAS and THI. Patients whose preoperative ABG was less than 15 dB showed no improvement in postoperative tinnitus using VAS (*p* = 0.889) and THI (*p* = 0.802). However, patients whose preoperative ABG was more than 15 dB showed statistically significant improvement in postoperative tinnitus using VAS (*p* < 0.01), and THI (*p* = 0.016) (Table 4). The postoperative Air Conduction (AC) and BC thresholds were significantly improved (Table 5). Table 6 shows significant postoperative changes in ABG and tinnitus compared to preoperative status. In preoperative ABG of more than 15 dB group, 55 patients were evaluated for postoperative PTA. The mean preoperative ABG was 17.9 dB and mean postoperative ABG was 14.4 dB. The audiological outcome significantly improved (*p* < 0.01). In addition, postoperative tinnitus showed significant improvement than preoperative tinnitus using VAS (*p* = 0.006). However, the correlation between the reduction in VAS score and ABG (Fig. 1) or between reduction in THI score and ABG (Fig. 2) was not significant.

**Table 1:** Demography of patients.

Age	50.7 (11–78) years
Sex (M:F)	24:51
Lesion site (Rt:Lt)	41:34

**Table 2:** Perforation type and location of the patients.

Size	Location	Patients	Total
Small	Anterosuperior	7	29
	Anteroinferior	15	
	Posterosuperior	2	
	Posteroinferior	5	
Moderate	Anterior	21	32
	Posterior	2	
	Inferior	9	
Large	Central	11	11
Near total	Central	3	3

**Table 3:** Relationship between postoperative changes in tinnitus and preoperative BC status.

	BC less than 25 dB (*n* = 50)			BC more than 25 dB (*n* = 25)		
	Preop mean ± SD	Postop mean ± SD	*p*-Value	Preop mean ± SD	Postop mean ± SD	*p*-Value
VAS	2.7 (±1.4)	1.7 (±2.0)	0.008	3.7 (±2.3)	2.8 (±2.8)	0.015

Paired Student's *t* test.

BC, bone conduction; VAS, visual analog scale.

**Table 4:** Relationship between postoperative change in tinnitus and preoperative ABG status.

	Group A (*n* = 23)			Group B (*n* = 52)		
	Preop mean ± SD	Postop mean ± SD	*p*-Value	Preop mean ± SD	Postop mean ± SD	*p*-Value
VAS	2.8 (±2.3)	2.7 (±3.0)	0.889	3.1 (±1.5)	1.8 (±1.9)	<0.01
THI	18.6 (±23.6)	20.0 (±29.5)	0.802	17.7 (±21.4)	9.7 (±16.4)	0.016

Group A, preoperative ABG less than 15 dB; Group B, preoperative ABG more than 15 dB.

ABG, air-bone gap; VAS, visual analog scale; THI, tinnitus handicap inventory.

Paired Student's *t* test.

**Table 5:** Preoperative and postoperative change of AC, BC (*n* = 55).

	Preoperative	Postoperative	*p*-Value
AC	39.2 dB	32.6 dB	<0.01
BC	21.2 dB	18.2 dB	0.001

Paired Student's *t* test.

AC, air conduction; BC, bone conduction.

**Table 6:** Relationship between preoperative and postoperative ABG and change in tinnitus.

	Preop	Postop	*p*-Value
ABG	17.9	14.4	<0.01
VAS	3.0	2.1	0.006

Paired Student's *t* test.

ABG, air-bone gap; VAS, visual analog scale.

## Discussion

Subjective tinnitus is more common than objective tinnitus. It may be caused by an abnormal condition in the cochlea, cochlear nerve, ascending auditory pathway, or auditory cortex.^10^

Any reversible otological factor including COM must be treated. Since Helms^8^ suggested that the subjective tinnitus symptoms might be reduced after middle ear surgery in COM with tinnitus, few studies^7^, ^8^, ^9^, ^11^, ^12^, ^13^, ^14^ have shown the subjective improvement of tinnitus after middle ear surgery. However, the results varied depending on the surgery type or pathological status of the middle ear. In this study, we studied the relationship between the change in subjective tinnitus and tympanoplasty type I. Our results showed an improvement of tinnitus after tympanoplasty type I similar to other reports. Kim et al.^9^ reported that the mean improvement of the AC average correlated with the improvement in tinnitus significantly, but ABG or BC average showed no significant change in tinnitus. However, our results were not similar to that of Kim et al.^9^ In the present study, tinnitus improved after surgery regardless of the preoperative BC level of more or less than 25 dB. The relationship between postoperative improvement in ABG and reduction in tinnitus showed significance. However, the preoperative ABG of less than 15 dB showed no improvement in tinnitus. When the preoperative ABG was more than 15 dB, tympanoplasty was effective in improving tinnitus. This was not similar to Lima et al.’s report^7^ that hearing loss discomfort was greater than that caused by tinnitus in all the time points considered. In the present study, the patient whose preoperative ABG of less than 15 dB complained tinnitus mainly than hearing loss in the preoperative history taking. A previous report showed that tympanoplasty usually improved tonal threshold and led to favorable tinnitus results by restoration of middle ear mechanics.^15^ However, tympanoplasty is not effective for improvement of tinnitus in the preoperative milder hearing loss group (ABG less than 15 dB). Our results indicate that the possibility of improvement of tinnitus after tympanoplasty to be very low in COM with mild hearing loss (less than preoperative ABG 15 dB). Therefore, patients with COM with milder hearing loss may be recommended for tinnitus treatment using sound therapy or other methods after surgery during the preoperative interview. We also think that patients with milder hearing loss do not feel a significant subjective hearing gain after tympanoplasty. One reason could be that with milder hearing losses, tinnitus may create more problems in daily life compared to hearing loss.^16^

Del Bo and Ambrosetti^4^ suggested two mechanisms for how tinnitus can be improved by hearing restoration. First, an increased level of ambient noise perceived after hearing restoration induces partial or complete masking of tinnitus, and second, the changes in the auditory nervous system caused by the deprivation of stimulus can be reversed by appropriate sound stimulation. Improvement in tinnitus after tympanoplasty has been reported positively in the literature. Lima et al.^7^ showed that 83% of 23 patients, and Baba et al. showed that 55% of 324 patients showed improvement in tinnitus after surgery, but the previous reports did not investigate deeply the possible relationship between tinnitus and hearing. Recently, Kim et al.^8^ reported that restoration of AC threshold was one of the most important factors contributing to the improvement of tinnitus. In the present study, preoperative ABG is an important predictor of tinnitus outcome after tympanoplasty. To the best our knowledge, we have determined the role of preoperative ABG as a predictor for tinnitus outcome after tympanoplasty for the first time. Although there is a reduction in ABG by improved AC threshold after surgery, if the preoperative ABG is less than 15 dB, the patient cannot feel the attenuation of tinnitus. In the present study, although tinnitus was improved by a postoperative hearing gain in patients whose preoperative ABG was more than 15 dB, the correlation between the reduction in VAS or THI scores after operation and hearing improvement was not statistically significant. This was similar to previous reports.^7^, ^9^ Kim et al.^9^ explained that the reason behind the insignificant correlation between the improvement in tinnitus and hearing improvement was due to multi-factorial causes of tinnitus including emotional stability after tympanoplasty. The limitation of this study is the small sample size and retrospective design.

## Conclusion

We suggest that the preoperative ABG can be a predictor of tinnitus outcome after tympanoplasty in COM with tinnitus.

## Conflicts of interest

The authors declare no conflicts of interest.
